# Internalized stigma related to COVID-19 and its psychosocial and mental health correlates: a multicentric health facility based observational study from Nepal

**DOI:** 10.3389/fpsyt.2023.1276369

**Published:** 2024-02-14

**Authors:** Bigya Shah, Ananya Mahapatra, Uday Narayan Singh, Vilok Mishra, Sunil Kumar Daha, Rajan Pande, Madan Ratna Neupane, Anita Banjade, Chandra Bhal Khatik, Tej Bahadur K. C., Rajesh Kumar Mandal, Samjhana Pokharel, Rishi Gupta, Krishna Bahadur G. C.

**Affiliations:** ^1^Department of Psychiatry, Patan Academy of Health Sciences, School of Medicine, Patan Hospital, Patan, Nepal; ^2^Department of Psychiatry, Dr. Baba Saheb Ambedkar Hospital and Medical College, New Delhi, India; ^3^Department of Emergency Medicine, Narayani Hospital, Birgunj, Nepal; ^4^Department of Dermatology, Institute of Medicine, Maharajgunj, Nepal; ^5^Bardibas Hospital, Mahottari, Nepal; ^6^Department of Internal Medicine, Bheri Hospital, Nepalgunj, Nepal; ^7^Bankatawa Primary Health Care Centre, Banke, Nepal; ^8^Department of Pediatrics, Suny downstate Health Sciences University, Brooklyn, NY, United States; ^9^Rapti Provincial Hospital, Dang, Nepal; ^10^Gender Equality and Social Inclusion (GESI), Jagaran Nepal, Kathmandu, Nepal; ^11^Cognis Mindcare, New Delhi, India; ^12^Department of Community Health Sciences, Patan Academy of Health Sciences, School of Medicine, Patan Hospital, Patan, Nepal

**Keywords:** internalized stigma, anxiety, depression, stigma, COVID-19, Corona virus, mental health, Nepal

## Abstract

**Introduction:**

The COVID-19 pandemic has led to physical and psychological complications and social consequences in the form of illness-related stigma. This study aimed (1) to assess the sociodemographic and clinical variable, as well as COVID-19 related knowledge and perception of persons admitted for COVID-19/Suspected COVID-19 in Nepal, (2) to determine their levels of COVID-19- related internalized stigma, depression, and anxiety symptoms, and (3) to evaluate the correlates of COVID-19- related internalized stigma.

**Materials and methods:**

It was a cross-sectional exploratory study with a convenience sample of 395 participants (306 confirmed cases, 89 suspected cases) conducted between July–October 2020 in four health facilities in Madhesh and Lumbini provinces of Nepal. We used a semi-structured questionnaire to assess sociodemographic details, clinical information, COVID-19-related knowledge, perception, COVID-19-related internalized stigma, and the Hamilton Anxiety Depression Scale (HADS) in Nepali language. Descriptive statistics, correlation analyses, and linear regression analyses were performed. The level of statistical significance was considered at *p* < 0.05.

**Results:**

Around 23.3% of the patients had anxiety symptoms, 32.9% had depressive symptoms, and 20.3% had high COVID-19-related internalized stigma (mean ISMI score: 2.51–4.00). Linear regression analyses showed a significant positive association of COVID-19-related internalized stigma total score, with the following eight factors, i.e., no income in the past one month (*p* = 0.013), below average socioeconomic status (*p* = 0.004), anxiety symptoms (*p* = <0.001), depressive symptoms (*p* = <0.001), recent testing positive for COVID-19 (*p* = <0.001), involuntary admission (*p* = <0.001), prior experience of being in isolation and quarantine (*p* = 0.045), and those who blame others for COVID-19 (*p* = 0.025).

**Conclusion:**

COVID-19 survivors and suspects are vulnerable to symptoms of depression, anxiety, and COVID-19-related internalized stigma. For the first time from Nepal, our data suggests that COVID-19-related internalized stigma is associated with anxiety and depression symptoms, perceived below-average socioeconomic status, involuntary admission, prior experience of being in isolation and quarantine, recent COVID-19 positive report, self-blame, below-average socioeconomic status and no income in the past one month. Mitigating and preventing internalized stigma associated with a public health crisis such as COVID-19 is imperative by diagnosing and treating such mental health issues early and designing interventions and policies especially targeting vulnerable populations focusing on their economic background and socio-cultural beliefs.

## 1 Introduction

The Coronavirus disease 2019 (COVID-19) pandemic, since its emergence in December 2019 in Wuhan, China, has transformed from a global health emergency to a humanitarian crisis worldwide of unprecedented magnitude. Because of its high infectivity rate and widespread morbidity and mortality, nations worldwide were compelled to enforce lockdowns, quarantine, and isolation, leading to several adverse psychosocial and economic consequences. Moreover, lack of knowledge about the illness, misconceptions, health anxiety, public hysteria triggered by infodemics, led to a psychosocial crisis and adverse mental health consequences (1).

The COVID-19 pandemic has also resulted in stigmatizing attitudes and beliefs followed by discrimination toward those suffering from COVID-19, health workers, and first responders involved in their management. Stigma has different facets. It has been further observed that persons who have been diagnosed with COVID-19 may not only suffer societal discrimination, but they are also at risk of internalizing these beliefs, developing feelings of inferiority and self-anger, eventually applying to themselves and behaving as stigmatized individuals (for instance, thinking that the disease is their responsibility or that because of it, they may be dangerous to others) (2–4). This internalized stigma can lead to devaluation of self and generate emotions of self-prejudice, guilt, or shame, which would further affect their behavior and lead to adverse mental health consequences such as depression and anxiety (3, 4). Stigma hampers diagnosis, treatment, prevention, and control of diseases as individuals tend to hide their identity, avoid social interaction, and follow health guidelines and healthy preventive adaptive behaviors. Thus, internalized stigma affects the health of not just an individual but society at large (5, 6).

Yuan and colleagues conducted a systematic review and meta-analysis on the prevalence of stigma in infectious diseases, including COVID-19 in 2022 and estimated the prevalence of COVID-19 stigma (enacted stigma and perceived stigma) as 35% [95% CI, 26–44%] among the affected individuals, indicating COVID-19 is stigmatizing. People from lower education and lower and middle countries were more vulnerable to contagious disease-related stigma (7). Moreover, various factors increased infection-related stigma such as place of living, being minorities (due to ethnicity, sexual orientation, gender identity), having contagious infection, the lethality of the disease, social isolation due to quarantine and physical distancing, elderly age group, physical comorbidities, fear aggravating factors such as insufficient knowledge about illness, unavailability of effective treatment (8–13). Despite similarities in stigma in various infectious diseases, each disease had different features of stigma. Hence, it is pertinent to understand the factors contributing to stigma in each infectious disease outbreak separately so that specific interventions to prevent and eliminate stigma can be designed (11). Further, COVID-19 stigma affects both suspected and infected individuals and it is associated with particular race, religion, and occupations, such as healthcare professionals and police officers, minorities (migrant workers), lower education, and those working in quarantine hospitals (14, 15). However, research on internalized stigma is minimal worldwide (16–20). It is difficult to compare the studies due to methodological differences such as the use of heterogeneous instruments to measure internalized stigma. Therefore, it necessitates understanding COVID-19-related internalized stigma and its associated factors even more.

### 1.1 The COVID-19 situation in Nepal

The first case of COVID-19 was diagnosed on the 23rd of January 2020 in Nepal. The country underwent the first lockdown from 24 March to 21 July 2020, prohibiting domestic and international travel. The first wave of COVID-19 cases occurred from July 2020 to February 2021. But vaccination for COVID-19 began in Nepal only on 27 January 2021. The second wave occurred between the end of March 2021 and July 2021, which was the most devastating of all the three waves. For the second time, the country underwent lockdown for four months, i.e., from April 29, 2021 to Sep 1, 2021 (21–23). The third wave began from Dec 2021 to Jan 2022, with multiple surges in COVID-19 cases (24). Like most developing nations, Nepal has faced its share of adverse consequences of the COVID-19 pandemic on its economic and public health care systems. With a population of 29.16 million, as of July 28, 2023, the country has reported 1,003,382 COVID-19 PCR positive, 153,972 COVID-19 Antigen positive and 112,031 deaths (25, 26). Measures like nationwide lockdowns, social distancing, travel restrictions, 76.5% of the population vaccinated with double doses as of February 2023, and booster intake of about 27% as of December 2022 have been adopted in Nepal (27). COVID-19 has affected mental health in Nepal and worldwide (3, 28). Studies from Nepal have documented that people living in quarantine centers, COVID-19 survivors, those with lower socioeconomic status, healthcare workers, and especially nurses were found vulnerable to mental comorbidities, social stigma, and discrimination during the COVID-19 pandemic in Nepal (22, 29–32).

However, there has not been a study that has specifically explored the internalized stigma of COVID-19 among the Nepalese population to date. We conducted a multicentric health facility-based observational study from Nepal to study internalized stigma related to COVID-19 and its psychosocial and mental health correlates. We hypothesize a potential relationship between internalized stigma related to COVID-19 with psychosocial factors and depressive and anxiety symptoms in patients suffering or suspected to be suffering from COVID-19. The purpose of the present study was three-fold: 1) to assess the sociodemographic and clinical variables, as well as COVID-19-related knowledge and perception of persons admitted for COVID-19/Suspected COVID-19 in Nepal, 2) to determine their levels of COVID-19- related internalized stigma, depression, and anxiety symptoms, and 3) to evaluate the correlates of COVID-19- related internalized stigma.

## 2 Materials and methods

### 2.1 Participants and procedures

This study was designed as an exploratory cross-sectional observational study conducted in COVID-19 cases, and suspects admitted to Narayani Hospital, Birgunj, Narayani Temporary Special Corona Hospital, Birgunj of province two and Corona Special Hospital, Beljhundi, Dang, Bheri Hospital and Primary Health Centre, Bankatawa of province five in Nepal. The study was conducted over five months (July-Nov2020) during the first wave of the COVID-19 pandemic in Nepal.

The sampling method employed for the study was convenience sampling based on the set inclusion criteria. We included all patients fulfilling the following inclusion criteria: 1) COVID-19 suspects or cases admitted to Narayani Hospital, Birgunj, Narayani Temporary Special Corona Hospital, Birgunj, and Corona Special Hospital, Beljhundi, Dang, Bheri Hospital and Primary Health Centre, Bankatawa 2) aged between 18–65 years 3) not suffering from any severe co-morbid physical illness 4) willing to provide informed consent.

### 2.2 Sample size estimation

The reference study used for the purpose was the only study conducted in Nepal to evaluate the prevalence of anxiety and depression among COVID-19 patients (34.0 and 31.0%, respectively) until our study was designed (32). Assuming absolute error or precision at 0.05 and an expected non-response rate of 10%, the study’s sample size was estimated to be 380.

### 2.3 Data collection

The participants were recruited at the time of their discharge from their respective health facilities or within the first week of their discharge if missed, once they provided written consent. The interviews were in-person using WHO precautions and physical distancing or telephonic interviews. The discussions were followed by psychoeducation about COVID-19 and referral to psychiatrists if mental health issues were identified in the participants. Four hundred twelve participants were approached for the study; six refused to participate. 11 questionnaires were not filled and removed. A total of 395 complete questionnaires were used for the analyses.

### 2.4 Assessments

We collected the participant’s data using the following measures:

#### 2.4.1 Sociodemographic sheet

It included participants’ age, gender, marital status, religion, ethnicity, education, occupation, employment status, source of income, perceived socio-economic status, type of family, place of stay, living status, address, name of the health facility where admitted, method of admission, if was in quarantine/isolation facility before access in the current setting.

#### 2.4.2 Clinical profile sheet

It included the source of possible COVID-19, current COVID-19 status, symptomatic or not, history of medical illness, history of diagnosed psychiatric illness at present and in the past, substance use history, and pattern in the past month.

#### 2.4.3 Semi-structured questionnaire on COVID-19 related knowledge and perception

Questions were made about the local context, dealing with the perception of COVID-19 concerning its dangerousness, ideas of self-blame, situations where stigmatized the most, reasons for worry and knowledge about the cause of COVID-19, ways of its transmission, and recovery. The questionnaires were translated into Nepali using the WHO translation-back-translation methodology (33). Then, the research team reviewed the content. Further, out of the three questions related to knowledge about the cause of COVID-19, mode of transmission, and recovery from COVID-19 infection, if responses in at least two questions were correct, it was labeled adequate knowledge.

#### 2.4.4 COVID-19-related internalized stigma scale

**Figure 1:**
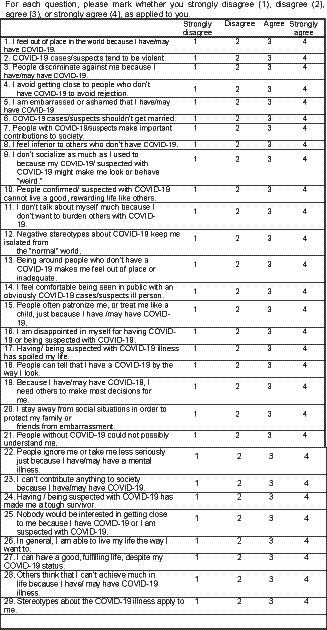
COVID-19 internalized stigma scale in English version. For each question, please mark whether you strongly disagree (28), disagree (13), agree (62), or strongly agree (31), as applied to you.

Since there was no scale to study internalized stigma to COVID-19 at the time of the study, the Internalized Stigma of Mental Illness (ISMI) scale was adapted for this study. It is a 29-item self-report that includes subscales: Alienation, Discrimination Experience, Social Withdrawal, Stereotype, Endorsement, and Stigma Resistance (34). Items were developed initially in participants diagnosed with a severe mental illness (SMI) (34). Answers were coded on the following 4-point Likert scale: 1 (strongly disagree), 2 (disagree), 3 (agree), and 4 (strongly agree). It has high internal consistency reliability (0.90), high test–retest reliability, and good evidence of construct validity (34). There are two methods of score interpretation (34). The 2-category method divides scores according to whether they are above or below the midpoint: 1.00–2.50 (does not report high internalized stigma) and 2.51–4.00 (reports high internalized stigma) (35). The score can also be used as a continuous variable. Permission was taken to use the author’s scale and translate it into Nepali. The English and Nepali versions of the scale are available in Figure 1.

The ISMI scale was adapted for this study by following a four-stage procedure similar to (36).

1. The first stage consisted of modifying the wording of the items so that these would be relevant for COVID-19 suspects and cases. Accordingly, “mental illness” was replaced by “Corona suspect and cases.”
2. The scale was translated to Nepali using the WHO translation-back-translation methodology.
3. The research team reviewed content through group discussions to reach a consensus regarding the face validity of the instrument.
4. Pilot testing to assess feasibility and comprehensibility among the participants was done among 40 cases and suspects in Bheri Hospital and PHC, Bankatawa. The items were comprehensible in all scales and semi-structured performa. Patients understood all points except item 10 on the internalized stigma scale. Hence, “like others” words were added for clarification in item 10.

#### 2.4.5 Hospital anxiety and depression scale

It is a psychiatric screening instrument to identify and assess anxiety and depression. Due to the absence of somatic symptoms, it is acceptable in hospital populations. The original English version of HADS contains 14 items in two subscales: anxiety (HADS-A) and depression (HADS-D), each with seven items (A1 to A7; D1 to D7) (37). Each item is rated on a four-point scale from 0–3 (3 indicating maximum symptom severity), and the scores are summed (five items on the depression subscale and one on the anxiety subscale are reversed before adding) (38). Each subscale, therefore, has a summed score with a potential range from 0 to 21. According to the original English version, a summed score of ≥11 on either subscale indicates caseness concerning the relevant psychiatric morbidity. Summed scores from 8–10 are “borderline” cases and 0–7 signifies the normal range (38). The Nepali version developed by Rijal and colleagues (24) will be used. Cronbach’s alpha was 0.76 for anxiety (HADS-A) and 0.68 for depression (HADS-D). All seven HADS-A items showed at least good item-to-factor correlations (range 0.44–0.74), and full construct validity was achieved for this subscale. Item-to-factor correlations for six HADS-D items were also at least acceptable (range 0.42–0.70); one thing (D4) had persistently low correlations throughout all trials, although construct validity was still satisfactory (39). Permission was taken to use the scale from the author.

### 2.5 Statistical analyses

Statistical analyses were performed using the Statistical Package for Social Sciences Version 26 (SPSS, Chicago, Illinois, USA). Descriptive statistics were calculated for socio-demographic and clinical characteristics and other relevant assessment instruments. As appropriate, data are presented as means and standard deviations (SD) or frequencies and percentages (%). A Spearman’s correlation analysis was performed to test the correlation between internalized stigma score, anxiety, and depression scale score. A combination of statistical association with the outcome, as well as theoretical importance, was considered while choosing predictors for the regression model. Among all theoretically important predictors, those with a univariate value of *p* <0.05 were considered for inclusion. If a variable was deemed theoretically very important, a value of *p* <0.2 was considered acceptable for inclusion. Care was taken not to include two theoretically redundant variables. Finally, a bidirectional stepwise selection was employed to choose the final set of variables for the multivariable model to determine the most parsimonious model. The level of statistical significance was kept at *p* < 0.05 for all the tests.

## 3 Results

### 3.1 Sociodemographic and clinical profiles

The demographic and clinical characteristics are summarized in Tables 1 2. The mean age of the participants was 32.5 (±11.0) years, 68.1% belonged to the 18–35 years age group, 26.1% were from Madhesi ethnicity, 70.4% were females, 71.4% were married, 65.1% were educated less than or up to class 10, and 36.5% were partially employed. Similarly, 55.2% did not have a source of income in the past one month, 56.2% perceived average socio-economic status, while majority stayed currently with family (78%), owned a house (74.2%). 20% of the participants were health workers, and 17% had involuntary admission to the health facilities (17%). 77.5% were COVID-19 cases and 22.5% were COVID-19 suspects. Most of them were from Province Five (81.8%), with no prior experience of staying in isolation or quarantine facilities before current admission (52.4%), did not get their COVID-19 test done recently (38.6%), asymptomatic (75.4%), with no history of medical illness (94.9%), and no current (99.2%), nor history of psychiatric illness (99.2%). The most common substance used in the past month was tobacco (23.8%), followed by alcohol (16.2%). Among the tobacco users, 45.8%used tobacco products mostly daily, but among alcohol users, they mostly used alcohol for more than half a week (78.1%).

**Table 1:** Sociodemographic of the participants (*N* = 395).

Sociodemographic variables	Frequency (%)	
Age categories	18–35 years 36–55 years 56 years and above	269 (68.1) 106 (26.8) 20 (5.1)
Age (in years)^a^	32.5 ± 11.0	
Gender	Male Female	117 (29.6) 278 (70.4)
Marital status	Unmarried Married Widow/widower	106 (26.8) 282 (71.4) 7 (1.8)
Religion	Hindu Buddhist Muslim	350 (88.6) 4 (1.0) 41 (10.4)
Ethnicity	Brahmin Kshettri Dalit Janajati Madhesi Others	54 (13.7) 97 (24.6) 65 (16.5) 64 (16.2) 103 (26.1) 12 (3)
Education completed	Less than and up to class 10 Above class 10	257 (65.1) 138 (34.9)
Health care workers		79 (20)
Current Employment status	Unemployed Partially employed Fully employed	115 (29.1) 114 (36.5) 136 (34.4)
Income source in past 1 month	Present Absent	177 (44.8) 218 (55.2)
Perceived Socioeconomic Status	Below average Average Above average	41 (10.4) 222 (56.2) 132 (33.4)
Current Stay	Alone With family With friends	75 (19.0) 308 (78.0) 12 (3.0)
Place of Stay	Own house Rent Hostel and quarter	293 (74.2) 83 (21.0) 19 (4.8)
Method of admission	Voluntary Involuntary	328 (83.0) 67 (17.0)
Names of centers	Narayani Hospital Dang Hospital Bheri Hospital PHC, Bankatawa	72 (18.2) 103 (26.1) 145 (36.7) 75 (19.0)
History of prior admission in isolation or quarantine center	Yes No	192 (48.6) 203 (52.4)

a = Mean ± SD.

**Table 2:** Clinical profile of the participants (*N* = 395).

Clinical variables		Frequency (%)
Suspects Cases		89 (22.5) 306 (77.5)
Recent COVID-19 test result	Positive Negative Report awaited Not done	90 (22.8) 133 (33.7) 22 (5.6) 150 (37.9)
Presentation	Symptomatic Asymptomatic	97 (24.6) 298 (75.4)
History of medical illness	Yes No	20 (5.1) 375 (94.9)
*Type of Medical illness DM HTN Asthma Hypothyroidism Congenital Heart Disease Pulmonary TB		8 (40.0) 6 (30.0) 5 (25.0) 1 (5.0) 1 (5.0) 1 (5.0)
History of a diagnosed psychiatric illness	Yes No	3 (0.8) 392 (99.2)
Current diagnosed psychiatric illness	Yes No	3 (0.8) 392 (99.2)
Substance use profile		
	Yes [Frequency (%)]	No [Frequency (%)]
Tobacco products	94 (23.8%)	301 (76.2)
Alcoholic beverages	64 (16.2%)	331 (83.8)
Cannabis	8 (2.0%)	387 (98.0)
Sedatives	2 (0.5%)	393 (99.5)
Opioids/ Others	0 (0%)	395 (100.0)

Frequency of substance use			
	Daily	More than half a week	Less than half a week
Tobacco products (*N* = 94)	43 (45.8)	13 (13.8)	38 (40.4)
Alcoholic beverages (*N* = 64)	1 (1.6%)	13 (20.3)	50 (78.1)
Cannabis (*N* = 8)	–	2 (25.0)	6 (75.0)
Sedatives (*N* = 2)	–	–	2 (100.0)

* Multiple response questions.

### 3.2 COVID-19 infection-related knowledge, perception, and internalized stigma related to COVID-19

As shown in Table 3, more than half of the participants did not know that COVID-19 infection was caused by the COVID-19 virus (51.6%). The majority knew that COVID-19 was a communicable disease (97%), only two-fifths attributed contact transmission (40.8%), and one-twenty-fifth mentioned vehicle-borne transmission (3.3%). However, most reported air transmission as the mode of transmission (66.1%), and four people attributed it to even fate for getting infected with COVID-19. One-fourth assumed friends as the possible source of information. More than one-fifth attributed home remedies as the way to recover from the infection, and only one in ten participants reported that COVID-19 illness is self-limiting. Most reported COVID-19 as dangerous (70.6%), and most attributed complications and death to its dangerousness (46.1%). More than one-fifth had inadequate knowledge (21.3%) of COVID-19 infection, and more than one-fourth blamed self for being infected with COVID-19 (25.1%). The participants were primarily worried about illness and health complications (64.2%), followed by social stigma (35.8%), being in isolation and quarantine (27.7%), the health of the family (17.0%), financial aspects (6.3%), transmission to others (3.8%), infodemics (1.9%), and education, work, and future (1.9%). Most of the participants felt that they were mostly stigmatized on the day of the diagnosis (32.7%), followed by during isolation and quarantine (32.7%), when utilizing health services (4.3%).

**Table 3:** COVID-19 infection related knowledge, perception and stigma perceived due to COVID-19 (*N* = 395).

Variables		Frequency (%)
Causes of COVID-19 infection	COVID-19 virus Others Unknown	191 (48.4) 161 (42.0) 42 (10.6)
Is COVID-19 a communicable disease?	Yes No Unknown	383 (97.0) 7 (1.8) 5 (1.3)
*Modes of its transmission	Air Contact Vehicle borne Due to fate Unknown	259 (66.1) 150 (40.8) 12 (3.3) 4 (1.0) 32 (6.8)
Source of transmission of COVID-19	Family member Friend Neighbor Patients Do not know	34 (8.6) 99 (25.1) 68 (17.2) 10 (2.5) 207 (52.4)
*How can someone recover from COVID-19 infection?	Self-limiting Medical management Boosting immunity Better hygiene Separation from others Religion Positive mental health Unknown Home remedies	48 (10.4) 105 (22.7) 85 (18.4) 45 (11.4) 45 (9.7) 2 (0.4) 3 (0.6) 36 (7.8) 95 (20.5)
Is COVID-19 dangerous?	Yes No	279 (70.6) 116 (29.4)
COVID-19 infection related Knowledge	Adequate Inadequate	311 (78.7) 84 (21.3)
*Why is COVID-19 dangerous?	No Medical cure Complications and death Communicable to others No Vaccination Heard from others No idea	43 (10.9) 182 (46.1) 22 (5.6) 1 (0.3) 13 (3.3) 135 (34.2)
Self-blame for infected with COVID-19	Yes No	99 (25.1) 296 (74.9)
*Major concern	Illness and health complications Social Stigma Isolation and quarantine Health of the family Transmission to others Financial Infodemics Education, work & future	204 (64.2) 114 (35.8) 88 (27.7) 54 (17.0) 12 (3.8) 20 (6.3) 6 (1.9) 6 (1.9)
*Most stigmatizing situation faced by the patient since the pandemic	On the day of the diagnosis During isolation and quarantine Not felt stigmatized While utilizing health services In neighborhood Abroad When symptomatic Do not know	129 (32.7) 129 (32.7) 93 (23.5) 17 (4.3) 3 (0.8) 4 (1.0) 1 (0.3) 20 (5.1)

* Multiple response questions.

### 3.3 Measures of COVID-19- related internalized stigma, anxiety, and depression in participants

The mean HADS-anxiety scale score was 7.2 (±4.2), and the mean HADS-depression score was 7.0 (±4.8), where scores between 8 and 10 were considered “borderline” and a score of ≥11 on either subscale indicates caseness (Table 4). More than one-fifth of the participants were anxiety cases (23.3%) and one-third were depression cases (32.9%). The majority of them were classified as “normal” for both anxiety (53.2%) and depression (53.2%). The mean value of the COVID-19-related internalized scale score of the participants was 2.2 (±0.4), where the maximum scale score is 4. One-fifth had high internalized stigma (20.3%). Among the internalized stigma-specific subdomains, the participants scored the most on stigma resistance subscale (2.4 ± 0.4), followed by social withdrawal (2.3 ± 0.6), discrimination experience (2.2 ± 0.5), alienation (2.1 ± 0.6), and stereotype endorsement (2.0 ± 0.6).

**Table 4:** Measures of internalized stigma, anxiety, and depression in participants (*N* = 395).

Variables	Mean ± S.D. or Frequency (%)
HADS-A score	
Categories:	
Normal Borderline Anxiety cases	210 (53.2)
	93 (23.5)
	92 (23.3)
Mean ± SD	7.2 ± 4.2
*HADS-D Score*	
Categories	
Normal Borderline Depression cases	210 (53.2)
	55 (13.9)
	130 (32.9)
Mean ± SD	7.0 ± 4.8
*ISMI score*	
Severity	
No high stigma High Stigma	315 (79.7) 80 (20.3)
Mean ± SD	2.2 ± 0.4
Subtypes	
Alienation	2.1 ± 0.6
Stereotype endorsement	2.0 ± 0.6
Discrimination experience	2.2 ± 0.5
Social withdrawal	2.3 ± 0.6
Stigma resistance	2.4 ± 0.4

### 3.4 Correlation and regression analysis

Spearman’s correlation revealed significant positive correlations between the ISMI total score and HADS-anxiety score (ρ- 0.6, *p* < 0.001) and HADS-depression score (ρ- 0.5, p < 0.001) (Tables 5 6). Except for stigma resistance, the other four sub-domains of the COVID-19-related internalized scale were significantly associated with HADS-Anxiety and HADS-Depression scores. Stigma resistance was only primarily related to the HADS-Anxiety score. Further, linear regression analyses showed a significant positive association of internalized stigma total score with eight factors, i.e., no income in the past one month (*p* = 0.013), below average socioeconomic status (*p* = 0.004), anxiety symptoms (*p* = <0.001), depressive symptoms (*p* = <0.001), recent testing positive for COVID-19 (*p* = <0.001), involuntary admission (*p* = <0.001), prior experience of being in isolation and quarantine (*p* = 0.045), and those who blame others for COVID-19 (*p* = 0.025). The model predicted 49% of the variance.

**Table 5:** Spearman correlation analysis between internalized stigma, anxiety, and depression scores (*N* = 395).

		1	2	3	4	5	6	7	8
1	ISMI total score	1	–	–	–	–	–	–	–
2	ISMI alienation sub score	0.811*	**1**	**–**	**–**	**–**	**–**	**–**	**–**
3	ISMI stereotype endorsement sub score	0.849*	0.621*	**1**	**–**	**–**	**–**	**–**	**–**
4	ISMI Social Withdrawal Sub score	0.602*	0.421*	0.418*	**1**	**–**	**–**	**–**	**–**
5	ISMI discrimination experience sub score	0.799*	0.640*	0.616*	0.449*	**1**	**–**	**–**	**–**
6	ISMI stigma resistance sub score	−0.013	−0.171*	−0.038*	−0.456*	−0.127*	**1**	**–**	**–**
7	HADS anxiety sub score	0.6*	0.645*	0.448*	0.451*	0.446*	−0.211*	**1**	**–**
8	HADS depression sub score	0.511*	0.550*	0.416*	0.342*	0.385*	**−**0.079	0.755*	**1**

* Correlation is significant at the 0.001 level (2-tailed).

**Table 6:** Linear regression.

Dependent: Stigma Score		Mean ± SD	Coefficient (univariable)	Coefficient (multivariable)
Age	18–35 Years	2.2 ± 0.4	–	–
	>35 Years	2.2 ± 0.3	0.03 (−0.05 to 0.11, *p* = 0.442)	–
Gender	Male	2.2 ± 0.4	–	–
	Female	2.3 ± 0.3	0.07 (−0.01 to 0.15, *p* = 0.067)	–
Marital status	Married	2.2 ± 0.4	–	–
	Not Married	2.2 ± 0.4	0.03 (−0.05 to 0.11, *p* = 0.523)	–
Education	Above 10	2.2 ± 0.4	–	–
	Up to 10	2.2 ± 0.3	0.04 (−0.04 to 0.11, *p* = 0.365)	–
Employment	Employed	2.2 ± 0.4	–	–
	Unemployed	2.2 ± 0.4	0.02 (−0.06 to 0.10, *p* = 0.706)	−0.06 (−0.12 to 0.01, *p* = 0.096)
Income in past 1 Month	No	2.3 ± 0.4	–	–
	Yes	2.1 ± 0.4	−0.13 (−0.20 to −0.06, *p* < 0.001)***	−0.08 (−0.15 to −0.02, *p* = 0.013)*
Health care professional	No	2.2 ± 0.4	–	–
	Yes	2.1 ± 0.4	−0.16 (−0.25 to −0.07, *p* < 0.001) ***	–
Current place of residence	Own House	2.2 ± 0.4	–	–
	Hostel/ Quarter/Rent	2.2 ± 0.3	0.03 (−0.05 to 0.11, *p* = 0.507)	–
Perceived SES	Average/ Above Average	2.2 ± 0.4	–	–
	Below Average	2.4 ± 0.4	0.27 (0.15 to 0.38, *p* < 0.001)***	0.13 (0.04 to 0.23, *p* = 0.004)**
Current tobacco use	No	2.2 ± 0.4	–	–
	Yes	2.2 ± 0.4	0.01 (−0.07 to 0.10, *p* = 0.745)	–
Anxiety symptoms	No	2.1 ± 0.3	–	–
	Yes	2.6 ± 0.4	0.47 (0.40 to 0.54, *p* < 0.001)***	0.33 (0.26 to 0.40, *p* < 0.001)***
Depression symptoms	No	2.1 ± 0.3	–	–
	Yes	2.5 ± 0.4	0.38 (0.31 to 0.45, *p* < 0.001)***	0.18 (0.12 to 0.25, *p* < 0.001)***
Recent COVID-19 Positive result	No	2.2 ± 0.4	–	–
	Yes	2.3 ± 0.4	0.11 (0.02 to 0.20, *p* = 0.012)*	0.12 (0.06 to 0.19, *p* < 0.001)***
Isolation and quarantine	No	2.2 ± 0.3	–	–
	Yes	2.4 ± 0.4	0.20 (0.11 to 0.28, *p* < 0.001)***	0.07 (0.00 to 0.13, *p* = 0.045)*
Mode of hospital admission	Self	2.1 ± 0.3	–	–
	Forced	2.5 ± 0.4	0.41 (0.32 to 0.50, *p* < 0.001)***	0.21 (0.13 to 0.29, *p* < 0.001)***
Symptomatic at admission	No	2.2 ± 0.4	–	–
	Yes	2.3 ± 0.3	0.08 (−0.01 to 0.16, *p* = 0.072)	–
Knowledge of COVID-19	Inadequate	2.2 ± 0.3	–	–
	Adequate	2.2 ± 0.4	0.03 (−0.06 to 0.12, *p* = 0.466)	–
Considers COVID-19 dangerous	No	2.1 ± 0.3	–	–
	Yes	2.3 ± 0.4	0.18 (0.10 to 0.25, *p* < 0.001)***	–
Self-blame for COVID-19	No	2.2 ± 0.3	–	–
	Yes	2.1 ± 0.5	−0.11 (−0.19 to −0.02, *p* = 0.013)*	−0.07 (−0.13 to −0.01, *p* = 0.025)*

*p < 0.05.

**p < 0.01.

***p < 0.001.

## 4 Discussion

This study demonstrated the sociodemographic, clinical variables, and COVID-19-related knowledge and perception of persons admitted for COVID-19/Suspected COVID-19 in Nepal. It also assessed their levels of internalized stigma to COVID-19, depression and anxiety symptoms and evaluated the correlates of internalized stigma to COVID-19. The present study, conducted during the first wave of the pandemic, is the first study from Nepal that has attempted to delineate the correlates of internalized stigma related to COVID-19.

The majority of the participants in the present study belonged to the age group of 18–35, were women (70.4%) and married (71.4%). This result is in contrast to a study conducted with 441 participants living in 9 selected quarantine centers across the provinces of Nepal, wherein among the 441 participants, 180 (40.9%) were aged 20–29 years, majority of the respondents were males (426/441, 96.6%) and labor workers (90%, 395/441) (29). One of the reasons for the difference in the gender distribution of the sample could be that, in the latter study, the majority of the sample constituted returnee migrants and health workers who were more likely to be men.

In our present study, the majority of the participants were not educated beyond class 10. This finding was similar to the previous study, where only 2% of the participants had a higher education (>12^th^ Grade). A study in India, conducted in multiple centers like ours, had a comparable monthly income. However, the mean age of the participants was 38 years, which was higher than ours, the majority were males and educated above class 12 (40). In both studies, the majority of the participants knew about the cause of COVID-19 infection but a lesser number of participants knew about the mode of transmission in our study than in the Indian study. Even though Nepal and India share similar sociocultural backgrounds and both studies were conducted in the first wave of COVID-19, the discrepancies in the result of knowledge may be because the two provinces selected in our study are not the most developed provinces of Nepal. However, the seven states, which were selected in the Indian study, are one of the developed states with the majority of the participants from urban areas and with good education and monthly income. Further, the use of different questionnaires on knowledge related to COVID-19 in both studies makes it difficult to compare results. Similarly, two studies on self-stigma, the construct of our focus, conducted in Lebanon and China had similar profiles of participants like ours.

The majority of participants in China had similar educational profiles, perception of socioeconomic, and health status, and most lived with their families. But they were mostly males and the mean age of the participants was 42.2 years (20). The majority of the Lebanese population were females with low or no income like ours. However, unlike ours, majority were singles, highly educated and only 10% were COVID-19 positive (16).

In our study, more than one-fifth of the participants were found to be suffering from anxiety (23.3%), and one-third were found to be suffering from depression (32.9%). This was significantly more than that reported from the previous study conducted in quarantine center in Nepal, where around 13.6% (60/441) of individuals kept in the quarantine centers were suffering from depression and 20.9% (92/441) of respondents were suffering from anxiety (29). However, it is important to note that, these respondents were not all cases or suspected cases of COVID-19 and were residing in quarantine centers because they were returnee migrants. The findings of the prevalence of depressive and anxiety symptoms are in sharp contrast to the literature among other studies conducted in Nepal (41), India (42, 43) and Korea (44). The differences may be because of heterogeneity in instruments used to measure anxiety and depression, time frames in which research was carried out, patients’ profiles- whether asymptomatic or recovered, general population or health care workers, etc.

During the COVID-19 pandemic, people have experienced stigma related to COVID-19 illness in various spectra: social stigma (16, 19, 45, 46) perceived stigma (15, 18–20, 29, 40, 42, 44, 47–50) and internalized stigma (15–20). The relationship between social and internalized stigma has been widely discussed (2, 51–53). The socio-cognitive model of internalized stigma in mental illness explains the cognitive processes involved in its development from social stigma: an individual is aware of the negative stereotype of the health condition and holds their attitude toward the state (stereotype awareness and social stigma), then as he agrees with the assumption (agreement) and eventually applies those stereotypes to themself (application) (2, 53). Internalized stigma has been consistently linked with poor outcomes such as poor self-esteem, severe psychiatric symptoms, and poor recovery from the health condition (51, 52). But very few studies have studied internalized stigma or self-stigma related to COVID-19 (15–20). In our study, about one-fifth (20.3%) of the participants admitted for COVID-19 had a high internalized stigma to COVID-19. This proportion is in sharp contrast to the higher proportion of self-stigma in an online survey conducted on the Lebanese population (65.9%) using the Self-Stigma Scale (16) and the lower prevalence of internalized shame of 2.7% in Chinese population (20). The latter study used the Social Impact Scale in which overall stigma was 12.9% (20). The difference in participants’ profiles as described above, methodologies, and scales for measuring internalized stigma explain the variation in the result. Therefore, it warrants the need to study internalized stigma among people with COVID-19 systematically. It requires excellent concern, attention, and awareness at national and global levels.

Our study reported that those who recently tested positive for COVID-19, involuntarily admitted, blamed others for COVID-19, with below average perceived socioeconomic status, no income in the past month, and prior experience of being in isolation and quarantine were significantly associated with internalized stigma to COVID-19. A growing body of literature has also suggested various other societal, structural, and personal factors aggravating the stigma of COVID-19. To name a few, fear and blame of transmitting the infection to others and being responsible for other’s deaths, social exclusion and social distancing during isolation and quarantine, physical violence and abuse, mental health issues, loss of livelihood, insensitive treatment by health care professionals, false information about COVID-19, social media as source of information, involvement of police in contact tracing and isolation, and legitimization of segregation by forced public health interventions, poor educational status, social support, and income have been widely discussed in the qualitative studies (18, 45, 48) and quantitative studies (16, 17, 19). However, as we have extensively assessed psychosocial factors associated with internalized stigma, similar work has not been carried out in other studies. In our research, we have identified additional factors positively associated with internalized stigma to COVID-19, i.e., involuntary admission and recent COVID-19 positive test report result. People admitted to hospitals and quarantine centers often share uncertainty, health concerns, boredom, and bad experiences in the form of insensitive behavior by staff and other colleagues and poor management in Nepal (29, 54) and elsewhere (15, 43, 45). So, forceful isolation and quarantine as a public health intervention acted as a double- edge sword and increased public stigma toward those with the illness (55). This could be why most of the participants in our study felt stigmatized on the day of the diagnosis and during isolation and quarantine. Also, in our research, internalized stigma was positively associated with having recent COVID-19 positive test results. Such a result can be easily explained by the same effect of isolation or quarantine for fear of being contagious. Hence, it can lead to more social withdrawal, discrimination, and internalized stigma among the cases and suspects. Additionally, in our study, other social and clinical factors contributed to internalized stigma, highlighting their roles as risk factors for internalized stigma. Hence, when they are timely identified, we can take measures to prevent and manage the internalized stigma of COVID-19. However, due to the different scales to measure internalized stigma and methodologies used while conducting the research, it is a great challenge to study their correlates among the available studies (56).

Internalized stigma to COVID-19 was significantly positively associated with depression and anxiety symptoms in our study. The findings are similar to other studies (17, 42, 44). In a pandemic, uncertainty about the future can result in anxiety (57). Social discrimination, exclusion, fear of rejection and abandonment lead to a negative appraisal of oneself and result in anxiety (58). Further, such concerns about a disease can result in negative behaviors toward others and a higher stigma (16), Similarly, the relationship between depression and stigma is based on several theoretical models of stigma and infectious disease or disability (59–63). The key features of depression are reduced self-esteem, ideas of guilt, and self-blame, which are also components of internalized stigma (64, 65). Depression can impair a person’s judgment and make a person agree and endorse negative stereotypes. Those with mental comorbidities have or tend to perceive poor social support and avoid interaction due to anhedonia. Such isolation and poor socioeconomic status reduce their sense of belonging to the group; they feel more alienated, discriminated against due to their condition, and socially withdrawn (66). Moreover, poor socioeconomic status and socio-occupational functioning, as seen in those with mental comorbidities, delay recovery and treatment-seeking, resulting in more social withdrawal, alienation, and discrimination (66).

The stigma of COVID-19 has led to difficulty in contact tracing as people fear disclosing the infection (18). Such people delay treatment seeking or not adhering to treatment, leading to medical complications. Also, they become more vulnerable to mental illnesses. Among healthcare professionals, such self-stigma toward their condition makes them demotivated toward their profession (66). Therefore, treatment and management of COVID-19 illness and the health system suffer due to stigma due to COVID-19. Hence, mitigating the internalized stigma of COVID-19 at the multidisciplinary level and among stakeholders such as the government, the public, healthcare providers, and religious leaders is of utmost importance (26). Early screening and identification of people for mental health issues such as anxiety and depressive symptoms with risk factors, especially among those who are socioeconomically disadvantaged, under isolation or quarantine repeatedly, COVID-19 positive test reports despite isolation and quarantine, and involuntarily admitted, and their timely treatment can reduce how they internalize stigma to COVID-19. Providing some financial support to those with poor socioeconomic status, employment sick leave, access to testing, and health insurance may address social inequalities and reduce stigmata. Similarly, home isolation may be encouraged rather than involuntary admission in mild cases. Attention to human rights by public health authorities and hospitals in COVID-19 wards is necessary. Providing clear information and addressing myths about COVID-19 can lead to reduced stigma (68). Anti-stigma education for the public, health professionals, cases, suspects, and their families is paramount. There exists the role of social support in moderating the effects of internalized stigma to COVID- (17). So, social support in the form of instrumental support (e.g., task assistance), informational support (e.g., guiding copying or problem-solving), and emotional support should be encouraged during such a pandemic.

We are aware of our limitations. It was a cross-sectional study conducted in only two provinces of Nepal during the first wave of the COVID-19 pandemic and only admitted COVID-19 confirmed or suspected persons were included. Hence, there are questions about the generalizability of the findings in the Nepalese population or other population profiles. Moreover, the result is not applicable to other time frames and at the current moment since COVID-19 is no longer a pandemic. Now that COVID-19 vaccines are available, we cannot verify the causal relationship of COVID-19 stigma-related variables. We used the ISMI scale for measuring internalized stigma to COVID-19 illness. Though it was translated and adapted using the WHO translation method and there was no scale to measure internalized stigma to COVID-19, the scale is not validated for the COVID-19 confirmed or suspected persons. There was no clinical diagnosis of anxiety and depression used. Though the HADS scale is valid and reliable for measuring anxiety and depressive symptoms, both the HADS scale & substance use history were self-reported. The study also had certain potential biases, such as convenience sampling method, psychosocial stressors, social and perceived stigma, unrecognized medical illnesses, social support, personality factors, and undiagnosed psychiatric comorbidities by clinicians. Therefore, future studies with a longitudinal study design addressing the potential biases can help us understand the COVID-19 stigma-related variables in a better way.

## 5 Conclusion

A significant proportion of COVID-19 survivors & suspects experience psychological morbidities such as depression and anxiety symptoms and internalized stigma. For the first time, our data suggest that internalized stigma of COVID-19 is associated with anxiety and depressive symptoms, perceived below-average socioeconomic status, involuntary admission, prior experience of being in isolation and quarantine, recent COVID-19 positive report, self-blame, below-average socioeconomic status and no income in the past one month. Therefore, it is imperative to diagnose and treat mental health issues early. The interventions, practices, guidelines, and public health policies should target vulnerable populations with a focus on their economic background and socio-cultural beliefs to mitigate and prevent internalized stigma related to COVID-19 in isolation and quarantine facilities.

## Data availability statement

The raw data supporting the conclusion of the article will be made available. Any inquiry can be directed to the corresponding author.

## Ethics statement

This study involving human participants were reviewed and approved by National Health Research Council, Nepal (registration number: 436/2020). The participants provided their written informed consent to participate in the study.

## Author contributions

BS: Conceptualization, Data curation, Formal analysis, Methodology, Project administration, Supervision, Validation, Visualization, Writing – original draft, Writing – review & editing. AM: Conceptualization, Formal analysis, Methodology, Validation, Visualization, Writing – original draft, Writing – review & editing. US: Conceptualization, Methodology, Project administration, Supervision, Writing – original draft. VM: Conceptualization, Methodology, Project administration, Software, Writing – original draft, Writing – review & editing. SD: Methodology, Project administration, Writing – original draft, Writing – review & editing. RP: Project administration, Supervision, Writing – review & editing. MN: Methodology, Project administration, Supervision, Writing – review & editing. AB: Methodology, Visualization, Writing – original draft. CK: Methodology, Project administration, Supervision, Writing – review & editing. TK: Methodology, Project administration, Supervision, Writing – review & editing. RM: Methodology, Project administration, Supervision, Writing – review & editing. SP: Visualization, Writing – review & editing. RG: Formal analysis, Visualization, Writing – review & editing. KG: Formal analysis, Visualization, Writing – review & editing.
